# Prevalence and Management of Cancer of the Rectal Stump after Total Colectomy and Rectal Sparing in Patients with Familial Polyposis: Results from a Registry-Based Study

**DOI:** 10.3390/cancers14020298

**Published:** 2022-01-08

**Authors:** Gaia Colletti, Chiara Maura Ciniselli, Stefano Signoroni, Ivana Maria Francesca Cocco, Andrea Magarotto, Maria Teresa Ricci, Clorinda Brignola, Clara Bagatin, Laura Cattaneo, Andrea Mancini, Federica Cavalcoli, Massimo Milione, Paolo Verderio, Marco Vitellaro

**Affiliations:** 1 Department of Surgery, Colorectal Surgery Unit, Fondazione IRCSS Istituto Nazionale dei Tumori, 20133 Milan, Italy; gaia.colletti@istitutotumori.mi.it (G.C.); marco.vitellaro@istitutotumori.mi.it (M.V.); 2 General Surgery Residency Program, University of Milan, Via Festa del Perdono 7, 20122 Milan, Italy; 3 Unit of Bioinformatics and Biostatistics, Department of Applied Research and Technological Development, Fondazione IRCCS Istituto Nazionale dei Tumori, 20133 Milan, Italy; chiara.ciniselli@istitutotumori.mi.it (C.M.C.); clara.bagatin@istitutotumori.mi.it (C.B.); paolo.verderio@istitutotumori.mi.it (P.V.); 4 Unit of Hereditary Digestive Tract Tumours, Department of Surgery, Fondazione IRCCS Istituto Nazionale dei Tumori, 20133 Milan, Italy; mariateresa.ricci@istitutotumori.mi.it (M.T.R.); clorinda.brignola@istitutotumori.mi.it (C.B.); 5 Department of General Surgery, Whipps Cross University Hospital, London E11 1NR, UK; i.cocco@nhs.net; 6 Diagnostic and Surgical Endoscopy Unit, Fondazione IRCSS Istituto Nazionale dei Tumori, 20133 Milan, Italy; andrea.magarotto@istitutotumori.mi.it (A.M.); andrea.mancini@istitutotumori.mi.it (A.M.); federica.cavalcoli@istitutotumori.mi.it (F.C.); 7 First Pathology Division, Department of Diagnostic Pathology and Laboratory, Fondazione IRCSS Istituto Nazionale dei Tumori, 20133 Milan, Italy; Laura.Cattaneo@istitutotumori.mi.it (L.C.); massimo.milione@istitutotumori.mi.it (M.M.)

**Keywords:** rectal stump cancer, FAP, hereditary syndrome, colorectal surgery, prevention, total colectomy, ileo-rectal anastomosis

## Abstract

**Simple Summary:**

Prophylactic total colectomy with ileo-rectal anastomosis (IRA) is the surgical approach that guarantees a better quality of life compared to proctocolectomy and ileo-anal anastomosis (IPAA) in familial adenomatous polyposis (FAP) patients. However, previous studies have warned about the high risk of cancer of the rectal stump, especially considering the young age of these patients. This is a retrospective study whose aim was to assess both clinical and surgical features of patients who developed cancer of the rectal stump. The data reported show that IRA is a safe approach from an oncological perspective. Since early tumors of the rectal stump may be easily detected via endoscopic or minimally invasive approaches, strict surveillance is necessary.

**Abstract:**

Background: The balance between quality of life and colorectal cancer risk in familial adenomatous polyposis (FAP) patients is of primary importance. A cut-off of less than 30 polyps under 1 cm of diameter in the rectum has been used as an indication for performing ileo-rectal anastomosis (IRA) in terms of lower rectal cancer risk. This study aimed to assess clinical and surgical features of FAP patients who developed cancer of the rectal stump. Methods: This retrospective study included all FAP patients who underwent total colectomy/IRA from 1977 to 2021 and developed subsequent rectal cancer. Patients’ features were reported using descriptive statistics by considering the overall case series and within pre-specified classes of age (<20, 20–30, and >30 years) at first surgery. Results: Among the 715 FAP patients, 47 (6.57%, 95% confidence interval: 4.87; 8.65) developed cancer in the rectal stump during follow-up. In total, 57.45% of the population were male and 38.30% were proband. The median interval between surgery and the occurrence of rectal cancer was 13 years. This interval was wider in the youngest group (*p*-value: 0.012) than the oldest ones. Twelve patients (25.53%) received an endoscopic or minimally invasive resection. Amongst them, 61.70% were Dukes stage A cancers. Conclusions: There is a definite risk of rectal cancer after total colectomy/IRA; however, the time interval from the index procedure to cancer developing is long. Minimally invasive and endoscopic treatments should be the procedures of choice in patients with early stage cancers.

## 1. Introduction

Familial adenomatous polyposis (FAP) is an autosomal dominant inherited condition associated with germline pathogenetic variants on the *APC* gene, characterized by the development of tens to thousands of adenomas in the large bowel starting from the second decade of life. It is strongly associated with colorectal cancer (CRC) risk [1,2]. Other hereditary familial polyposis syndromes like *MUTYH*-associated polyposis, an autosomal-recessive disorder, and the recently defined NAMP (non-*APC/MUTYH* polyposis) are indeed linked to a milder phenotype [3,4].

Considering that CRC risk during the lifetime of FAP patients is near 100%, prophylactic total colectomy with or without rectal sparing is recommended as the only chance of avoiding cancer in the large bowel [5]. Duodenal or ampullary carcinoma and thyroid carcinoma are the most frequent extracolonic malignant lesions occurring in FAP patients. Cancers of the pancreas, liver, brain, and adrenals have also been reported [6,7,8,9]. Furthermore, it is acknowledged that the risk of desmoid tumors is higher than in the general population and could be enhanced by surgery [10,11].

FAP guidelines [1,12] do not clearly define an age cut-off for when patients should have surgery. Most of these patients undergo surgery between the second and third decades [5] of life for either symptoms linked to the presence of colon cancer and/or for prophylactic intent in relatives of FAP patients (call-up). Patients undergoing surgery at a young age, for example, the “call-up” group, have a high quality of life’s expectancy, hence it should be of primary importance to maintain a high quality of life (QoL) whilst providing good surveillance for CRC [13,14,15].

Total colectomy with ileo-rectal anastomosis (IRA) is the procedure of choice that guarantees a better QoL and functional outcome compared to proctocolectomy with ileal pouch-anal anastomosis (IPAA) [16,17]. In addition, the laparoscopic approach is a highly feasible alternative for prophylactic treatment of FAP, reducing postoperative desmoid tumor incidence, and is more accepted by younger patients [18,19]. On the other hand, for patients treated with colectomy/IRA, the cumulative risk of developing rectal cancer increases with time. Thus, a very careful and intensive surveillance program for these patients is needed [18,20]. 

Traditionally, for surgical management of polyposis syndromes at the Fondazione IRCCS Istituto Nazionale dei Tumori in Milan (National Cancer Institute of Milan)-Italy, a cut-off of less than 30 polyps in the rectum smaller than 1 cm is used as an indication for performing IRA [18] to lower cancer risk. This cut-off is easier to reach in classical FAP, with its left-sided predominance of polyp distribution, especially at older ages, than in milder phenotypes of polyposis syndromes (MAP and NAMP) [21]. To date, over 4000 families have been enrolled in the Hereditary Digestive Tract Tumors Registry at our center due to genetic tumor risk syndromes. These include over than 900 families affected by familial polyposis.

The aim of this study was to describe the features and management of FAP patients that developed cancer of the rectal stump after total colectomy and IRA. Using data from our institutional database, we aimed to highlight the critical steps in its management.

## 2. Materials and Methods

### 2.1. Case Series

This study included all FAP patients from the Hereditary Digestive Tract Tumors Unit database of the National Cancer Institute of Milan, that were treated with total colectomy and IRA since 1977 and developed a cancer of the rectal stump later on. This is an update of a previously published cohort from Bertario et al. [22]. Patients treated with proctocolectomy and ileopouch anastomosis (IPAA) or patients not able to follow the follow-up directions were excluded. For each patient, we collected: demographic characteristics (i.e., age, gender), genetic information (i.e., genetic test, probands, *APC* pathogenic variant), and surgical and pathological data of the first surgery and details of the second treatment. 

All patients in the registry were either diagnosed with germline mutations in the *APC* and/or *MUTYH* gene, belonged to a family in which the gene was previously tested, or affected by adenomatous polyposis with no identified *APC/MUTYH* variant, i.e., NAMP. The first subjects in a family that received genetic counseling and/or testing were defined probands, while call-ups were found to have FAP after diagnosis of FAP in relatives and/or during screening.

The patients followed up after total colectomy/IRA underwent flexible sigmoidoscopy every 6–12 months or more frequently in cases of high-grade dysplasia (HGD) oesophagogastro duodenoscopy (OGD) according to the Spigelman classification (at least every 4 years) and annual abdominal ultrasound. Patients underwent further investigations when symptomatic.

Histological classifications for colon and rectal cancer cases were reported according to the Dukes staging system. Follow-up details were also recorded. The study was approved by the Ethical Committee (#INT 184-21).

### 2.2. Statistical Analysis

Patients’ characteristics were summarized using basic descriptive statistics by considering the overall case series and within pre-specified age classes (<20, 20–30, and >30 years) at the first surgery. Distribution tables were computed for categorical data, whereas medians and interquartile range (IQR) were estimated for continuous data. The association between variables was assessed by using the nonparametric Kruskal–Wallis, chi-squared, and Fisher exact test according to the type of variables. Overall survival (OS) was calculated as the time from the second surgery to death due to any cause and the pattern of OS was estimated using the Kaplan–Meier method. All statistical analyses were carried out with SAS (Version 9.4; SAS Institute, Inc., Cary, NC, USA) and R software.

## 3. Results

Between 1977 and June 2021, data from 715 patients with FAP referred to the Unit of Hereditary Digestive Tract Tumors at National Cancer Institute of Milan were prospectively collected in the Hereditary Colorectal Tumor Registry after surgery. All these patients underwent total colectomy with IRA and underwent follow-up according to the institution’s surveillance protocol. Among them, 47 patients (6.57%, 95% CI: 4.87; 8.65) developed subsequent cancer in the rectal stump. 

Fourteen patients (29.79%) underwent a prophylactic total colectomy before the age of 20, while 14 patients between the second and third decades. The remaining 19 (40.43%) patients underwent surgery after the age of 30. There were 27 male patients (57.45%), with the highest M/F ratio in the 20-30 years age group (Table 1). 

**Figure 1:**
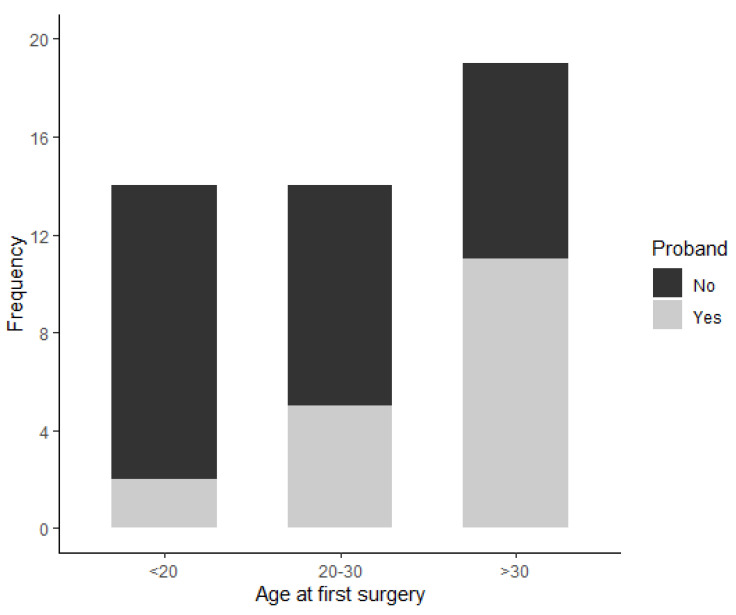
Shows the surgical details. The histopathology report was of adenocarcinoma in 10 patients (21.28%). Cancers were diagnosed in the early stages. In fact, nine patients had a Dukes’ stage A and B and one patient had a Dukes’ stage C adenocarcinoma as reported in Table 2.

Eighteen patients were probands (38.30%), while the other 29 (61.70%) were defined as ‘’call-ups’’. The proportion of probands was statistically correlated with age classes (Chi-square *p*-value: 0.04), with the highest percentage in the >30 years old age group and the lowest in the <20 years old age group (Figure 1). A genetic assessment was performed in 93.62% of the patients, leading to the identification of a germline pathogenetic variant in the *APC* gene. The remaining three patients were all probands that underwent colectomy/IRA before 1990. One patient was considered as NAMP because the genetic test did not show any *APC/MUTYH* pathogenetic variant. The remaining two did not have a genetic test performed and the diagnosis of FAP was made on the basis of clinical criteria (i.e., >100 adenomas in the large bowel). Regarding the *APC* pathogenic variant, 21.28% of the patients had the p.Glu1309Aspfs*4 mutation (which is associated with higher risk of cancer). Of note, 80% of them were <20 years old at the first surgery (Fisher p-value: 0.001). The remaining two patients were 20–30 and >30 years old at the first surgery. 

**Figure 2:**
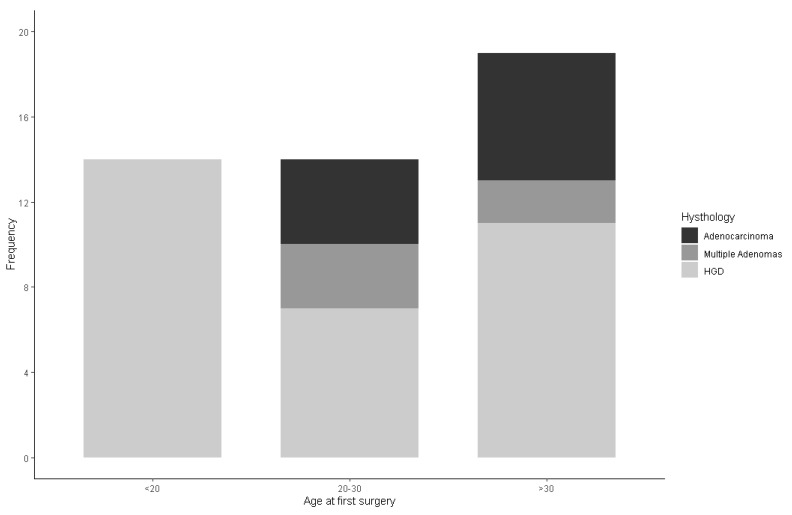
Bar charts representing the distribution of the baseline histology according to the three age classes. On the x-axis are reported the three age classes and on the y-axis the number of subjects according to baseline histology.

Cancer was found in the elder groups and none of the patients belonging to the youngest group of age were diagnosed with CRC or tubular villous adenoma with low-grade dysplasia (LGD) (Fisher *p*-value: 0.02, Figure 2).

The overall median interval between surgery and the diagnosis of cancer of the rectal stump was 13 years (IQR: 9–18 years) as shown in Table 3. This interval was wider in the youngest group (median: 20, IQR: 13–24 years) than in the older ones (median 10.5 and 11 years in the 20–30 and >30 years age classes, respectively) (Kruskal–Wallis test *p*-value: 0.012). 

Surgery was the treatment of choice for cancer of the rectal stump for the majority of patients (35, 74.47%). Four of them underwent a minimally invasive approach with transanal minimally invasive surgery (TAMIS), and amongst them, only one had to undergo further surgical treatment due to histologically positive margins.

**Figure 3:**
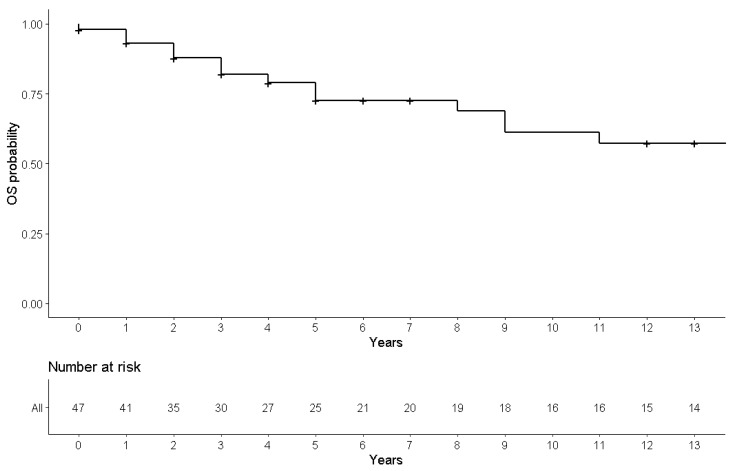
Thirteen-year OS probability in FAP patients from rectal cancer treatment.

The rest of the patients underwent either proctectomy with defunctioning ileostomy (38.30%) or ileorectal resection (27.66%). Twelve (25.53%) patients with cancer in the rectal stump did not receive surgical treatment. Eight cases were treated with an endoscopic mucosectomy (all of them were Duke’s stage A cancers), while four cases underwent palliative chemotherapy or chemo-radiotherapy. At an overall median follow-up of 13 years (IQR: 2–17), 33 were alive and 14 died, 13 due to rectal cancer progression and/or metastasis while one died due to non-cancer-related causes (Figure 3).

## 4. Discussion

In our study population, the prevalence of cancer of the rectal stump was 6.57% with the median time frame between total colectomy with IRA and treatment of the rectal cancer being 13 years. This data suggests a slow adenoma to carcinoma progression, as recently stated in a study by St Mark’s Hospital based on their Polyposis Registry Population [23]. This one reports only one case of cancer of the rectal stump out of 191 patients after a median follow-up of 8.6 years. Our results are consistent with theirs, especially considering that we included a high number of patients with the *APC* pathogenic variant (91%).

Another study from Pasquer et al., with a longer median follow-up time (17 years versus 13 years) and a smaller patient population (197 patients treated with total colectomy and IRA and 92 patients treated with proctocolectomy and IPAA), showed an incidence of cancer of the rectal stump of 6.1%. Their 25-year cancer-free survival was 96.3% in the IRA group and 98% in the IPAA group [24].

Here, three pre-specified age classes were identified, showing a statistically significant difference (*p*-value 0.012) between the median time from surgery and time of diagnosis of cancer of the rectal stump in the youngest group compared to the older ones (20–30 and >30 years age classes, respectively). These results are probably related to better management and surveillance of patients over the last two decades. Patient adherence to the surveillance protocol was in fact adequate probably because patients enrolled in the registry felt safer when followed up by a dedicated team. As recently shown, patients with a hereditary condition included in a regular surveillance program (registry) feel reassured by knowing that their surveillance is programmed by a team of experts [25].

It is acknowledged that FAP patients have a risk of developing rectal cancer after prophylactic total colectomy with IRA [20,22,26,27,28]. For this reason, it is extremely important that these patients receive a postoperative follow-up in tertiary referral centers where the surveillance protocol is well established. The risk of rectal cancer should not be underestimated and should be considered acceptable when associated with close endoscopic follow-up (FU) tailored for each patient. These patients should have an endoscopic FU every 6–12 months, but this should be more frequent in cases of high-grade dysplasia (HGD) to prevent locally advanced cancers in the rectal stump. Once diagnosed, cancer in the rectal stump should be appropriately assessed to establish the best therapeutic strategy. Staging should include a chest and abdomen CT scan with contrast to assess the presence of distant metastases and a MRI pelvis +/- endorectal ultrasound to assess the T and N stage of the tumor. Patients with local or distant metastatic disease should be discussed by a multidisciplinary team including a medical oncologist and radiotherapist to evaluate for neoadjuvant treatment. Early stage small rectal lesions could instead benefit from a minimally invasive approach, such as endoscopic excision or transanal resection. Huge tumors should instead be treated with a classical surgical approach, i.e., laparoscopic or open resection [29].

Kostenvuo et al. [20], in 2014, reported a study including 140 FAP patients, showing a cumulative risk of rectal cancer of 3% at 5 years, 4% at 10 years, 11% at 20 years, and 24% at 30 years after the first surgery. In total, 28% of the patients underwent a further proctectomy. They concluded that total colectomy/IRA should be the first choice of treatment only in patients with attenuated FAP (less than 100 polyps in the colon). However, they did not consider the presence of cancer at the time of the first surgery and *APC* mutation [30]. We believe that our indication to perform total colectomy with IRA when there are <30 polyps in the rectum considers a more specific subset of patients.

This study consolidates previous data from our registry [22]. This update was needed due to the fact that new minimally invasive approaches were introduced in this field and were offered to our patients during the last two decades, namely endoscopic or transanal excision.

Endoscopic follow-up is a lifelong part of patients with familial polyposis. It is evident that surveillance after the first surgery [31], especially for patients who underwent IRA, is crucial to limit rectal cancer risk. Moreover, endoscopic and clinical follow-up in tertiary centers could provide better outcomes in the management of these patients [32]. Endoscopic surveillance must fulfill high-quality endoscopic criteria including high-definition endoscopy with the possibility of electronic chromoendoscopy, and good rectal preparation. In addition to that, the importance of developing different strategies to reduce polyp burden and delaying cancer onset in FAP patients treated with IRA, such as chemoprevention with anti-inflammatory drugs and/or omega-3 supplements, has been recently shown [33]. New studies about diet and its key role in potentially reducing bowel inflammation and polyp burden in operated patients are promising and could contribute to finding a personalized therapeutic approach for FAP patients [34].

In this study, more than half of the patients (64%) developed an early stage rectal cancer (A or B, according to Dukes’ classification). According to these data, recent minimally invasive techniques, such as TAMIS [35] and multistage polypectomy with flexible sigmoidoscopy [23], could be considered the main and resolute approach in most cases of cancer of the rectal stump. Our approach is in line with that of the Center for Hereditary Colorectal Neoplasia of Cleveland Clinic Foundation (Cleveland, Ohio) [36], with, in our opinion, narrower initial selective criteria, such as, for instance, the genetic background of the single patient, together with the phenotype and the clinical features.

Iwama et al. [37] found that the longer the rectal stump, the higher the risk of developing rectal cancer: (10.3 ± 4.7 cm vs. 13.9 ± 5.3 cm), and this was reported to be statistically significant (*p* < 0.001). In patients with a short rectal stump, it is advisable treat cancer with minimally invasive techniques [38].

We, unfortunately, did not record neither the length of the rectal stump nor the distance of the tumor from the anal verge; this could be considered in future studies to also confirm the positive trend in OS of this subgroup of patients treated with minimally invasive techniques.

## 5. Conclusions

Considering the lifetime risk of cancer in different sites in FAP patients, maintaining patients’ high quality of life should be the key point of any treatment strategy, which should be customized according to the genetic and tumor clinical features. Rectal cancer risk after total colectomy/IRA is a real but potentially manageable risk. Minimally invasive or endoscopic treatment of cancer of the rectal stump is preferred in early stage cancers.

## Figures and Tables

**Table 1:** Baseline demographical and genetic characteristics.

Variable		<20 Years	20–30 Years	>30 Years	All
**Gender**	M	7 (50.00)	9 (64.29)	11 (57.89)	27 (57.45)
	F	7 (50.00)	5 (35.71)	8 (42.11)	20 (42.55)
**Proband**	Yes	2 (14.29)	5 (35.71)	11 (57.89)	18 (38.30)
	No	12 (85.71)	9 (64.29)	8 (42.11)	29 (61.70)

**Table 2:** Baseline surgical details.

Variable		<20 Years	20–30 Years	>30 Years	All
**Histology**					
	Multiple Adenomas	14 (100.00)	7 (50.00)	11 (57.89)	32 (68.09)
	HGD	-	3 (21.43)	21 (10.53)	5 (10.64)
	Adenocarcinoma	-	4 (28.57)	6 (31.58)	10 (21.28)
**Site (Adenocarcinoma)**					
	Left Colon	-	-	2 (33.33)	2 (20.00)
	Right Colon	-	2 (50.00)	-	2 (20.00)
	Rectosigmoid Junction	-	1 (25.00)	1 (16.67)	2 (20.00)
	Multicenter	-	1 (25.00)	3 (50.00)	4 (40.00)
**Stage (Adenocarcinoma)**					
	A	-	3 (75.00)	4 (66.67)	7 (70.00)
	B	-	-	2 (33.33)	2 (20.00)
	C	-	1 (25.00)	-	1 (10.00)

**Table 3:** Rectal stump surgical details.

Variable	Title	<20 Years	20–30 Years	>30 Years	All
**Time between first surgery and rectal ca. detection (years)**		20 (13–24)	10.5 (2–16)	11 (5–16)	13 (9–18)
**Stage**					
	A	7 (50.00)	8 (57.14)	14 (73.68)	29 (61.70)
	B	1 (7.14)	2 (14.29)	1 (5.26)	4 (8.51)
	C	5 (35.71)	3 (21.43)	4 (21.05)	12 (25.53)
	D	1 (7.14)	1 (7.14)	-	2 (4.26)
**Treatment**					
	Surgery	12 (85.71)	9 (64.29)	14 (73.68)	35 (74.47)
	No Surgery	2 (14.29)	5 (35.71)	5 (26.32)	12 (25.53)
**Treatment Details**					
	Proctectomy	7 (50.00)	5 (35.71)	6 (31.58)	18 (38.30)
	Ileorectal Resection	4 (28.57)	3 (21.43)	6 (31.58)	13 (27.66)
	TAMIS	1 (7.14)	1 (7.14)	2 (10.53)	4 (8.51)
	Endoscopic Mucosectomy	1 (7.14)	3 (21.43)	4 (21.05)	8 (17.02)
	RT/RT+CT	1 (7.14) *	2 (14.29)	1 (5.26)	4 (8.51)

* RT alone.

## Data Availability

Data are available on request.
